# Future climate change scenarios in Central America at high spatial resolution

**DOI:** 10.1371/journal.pone.0193570

**Published:** 2018-04-25

**Authors:** Pablo Imbach, Sin Chan Chou, André Lyra, Daniela Rodrigues, Daniel Rodriguez, Dragan Latinovic, Gracielle Siqueira, Adan Silva, Lucas Garofolo, Selena Georgiou

**Affiliations:** 1 Climate Change, Agriculture and Food Security Program, International Centre for Tropical Agriculture, Hanoi, Vietnam; 2 Tropical Agricultural Research and Higher Education Center, Turrialba, Costa Rica; 3 National Institute for Space Research, Cachoeira Paulista, SP, Brazil; 4 Alberto Luiz Coimbra Institute for Graduate Studies and Research in Engineering, Rio de Janeiro, RJ, Brazil; Universidade de Aveiro, PORTUGAL

## Abstract

The objective of this work is to assess the downscaling projections of climate change over Central America at 8-km resolution using the Eta Regional Climate Model, driven by the HadGEM2-ES simulations of RCP4.5 emission scenario. The narrow characteristic of continent supports the use of numerical simulations at very high-horizontal resolution. Prior to assessing climate change, the 30-year baseline period 1961–1990 is evaluated against different sources of observations of precipitation and temperature. The mean seasonal precipitation and temperature distribution show reasonable agreement with observations. Spatial correlation of the Eta, 8-km resolution, simulations against observations show clear advantage over the driver coarse global model simulations. Seasonal cycle of precipitation confirms the added value of the Eta at 8-km over coarser resolution simulations. The Eta simulations show a systematic cold bias in the region. Climate features of the Mid-Summer Drought and the Caribbean Low-Level Jet are well simulated by the Eta model at 8-km resolution. The assessment of the future climate change is based on the 30-year period 2021–2050, under RCP4.5 scenario. Precipitation is generally reduced, in particular during the JJA and SON, the rainy season. Warming is expected over the region, but stronger in the northern portion of the continent. The Mid-Summer Drought may develop in regions that do not occur during the baseline period, and where it occurs the strength may increase in the future scenario. The Caribbean Low-Level Jet shows little change in the future. Extreme temperatures have positive trend within the period 2021–2050, whereas extreme precipitation, measured by R50mm and R90p, shows positive trend in the eastern coast, around Costa Rica, and negative trends in the northern part of the continent. Negative trend in the duration of dry spell, which is an estimate based on evapotranspiration, is projected in most part of the continent. Annual mean water excess has negative trends in most part of the continent, which suggests decreasing water availability in the future scenario.

## Introduction

Central America region holds several countries (Honduras being the first, Nicaragua and Guatemala) within the top ten in the long term climate risk index [1] and is already suffering the effects of climate change as a result of historical warming and increased precipitation intensity trends [2]. Future climate scenarios also indicate general warming and reduced precipitation trends [3] with increased extremes [4]. The vulnerability to climate extremes in the region is high due to the strong dependence of the economy on agriculture and hydropower. Smallholder farmers are a vulnerable group in case of extreme changes of climate conditions [5].

The narrow land area of the continent across this region, where the distance between the Pacific and Atlantic Ocean can be less than a hundred kilometres is combined with complex topography in some areas that may reach the heights of about 4000 m in a few kilometres of horizontal distance. Long-term climate simulations using coarse horizontal resolution may have difficulty in describing surface conditions of this narrow continent. Therefore, high-resolution future scenarios are required to provide appropriate climate change information for impact, vulnerability and design of adaptation responses.

The effects of regional long-term variabilities are shown in the annual north-south displacement of the Intertropical Convergence Zone (ITCZ), the intensity of the subtropical high-pressure system over the Caribbean Sea, the strength of the trade winds and of the Caribbean Low-Level Jet (CLLJ) [6]. Central America has a well-defined rainy season from May to October [7]. However, the rainy season is characterized by two rainfall maxima (May/June and September/October) separated by a relative dry period (July/August) that is termed mid-summer drought (MSD) [8]. This bimodal distribution is more clearly defined over southwestern Mexico, Central America, and the eastern Pacific warm pool, where the ITCZ is active during summer [9]. The MSD has a socioeconomic importance because the agriculture and hydropower production are linked with seasonal cycle of precipitation. According to [10], CMIP5 models are capable of simulating the MSD over much of the Inter-Americas. [11] investigated how MSD may change in a warming climate projected by CMIP5 models. The results show that CMIP5 multi-model mean projects a strengthening of the MSD related to present climate similar to CMIP3 runs [12]. The region is also affected by El Nino variability.

The Atlas of projections included in the IPCC 5th Assessment Report [13] shows decrease in the median precipitation in most of Central America under the RCP4.5 scenario. These projections are generated by global climate models with strong agreement among the simulations for Central America. However, most of global climate models available from the CMIP5 have resolutions of about a few hundreds of kilometres and give little spatial detail of the climate changes in the region that features narrow continent, high mountains of steep slopes. [14] downscaled climate change scenarios to 50 km horizontal resolution using PRECIS in the region under A2 SRES scenarios [15] and showed the reduction of precipitation during both wet and dry periods. [4] used the MRI-AGCM3.1 model to downscale future scenarios with a global 20-km horizontal resolution model to estimate precipitation extremes. They showed an increase of the consecutive five-day accumulated precipitation exceeding 80% of Central America, which agreed with previous studies [16–18]. Studies using high resolution downscaling of climate change have shown some added value over the coarser model simulations [19]. Therefore, the main objectives of this work are to evaluate the high-resolution downscaling simulations over Central America and to assess climate change projections under RCP4.5 scenario. The uncertainty analysis and variability possible in the system are not carried out since this study is based on a single model projection.

## Methodology

The future climate scenario providing boundary conditions for the simulations is initially described, followed by the description of the Regional Climate Model that provides the downscaled simulations. The present climate, or baseline, is taken as the 30-year period between 1961 and 1990 and the future 30-year period between 2021 and 2050. The model evaluation presented is based on mean monthly and seasonal climate.

### The climate change scenario

The HadGEM2-ES model provides the boundary conditions [20]. This is a global model of earth system category, with horizontal resolution of about 200 km x 150 km. The detailed characteristics of this model are summarized in Table 9.A.1 in [21].

The model captures the general pattern of temperature and precipitation around Central America, ranking the 55^th^ position out of 107 global models evaluated by [22]. However, for nesting purposes, the quality of the state variables of HadGEM2-ES, such as three-dimensional wind, temperature, and moisture are more relevant than precipitation or surface temperature, as the latter variables do not drive the regional model.

The RCP4.5 scenario considers a future climate of medium to low emission of greenhouse gases to the atmosphere [23]. This scenario stabilizes radiative forcing at 4.5 W m^−2^ in the year 2100, which is about 650 ppm CO_2_-equivalent, without ever exceeding that value. Simulations extended until 2050 when the range in radiative forcing across RCPs is small compared to their dispersion in 2100 [24].

### The Regional Climate Model

The long-term climate simulations are produced by the regional climate version of the Eta Model [25,26]. The model has been used for weather forecasts [27], seasonal forecasts [28], and climate change studies [29–31] over South America. The eta coordinate [32] of the Eta model reduces the problem of calculation of horizontal pressure gradient near steep mountain regions. This is a common problem in numerical models. Therefore, the eta coordinate makes the model suitable to run over the regions aroung the Andes Cordillera. A description and refinement of the vertical coordinate was introduced further by [25]. Evaluation of the model long-term simulations [26,19] and assessment of future climate change projections [30,31,33] have been carried out over South America at different horizontal resolutions and different domains. Assessment of climate change in South America under RCP4.5 and RCP8.5 scenarios are shown in [31].

**Fig 1:**
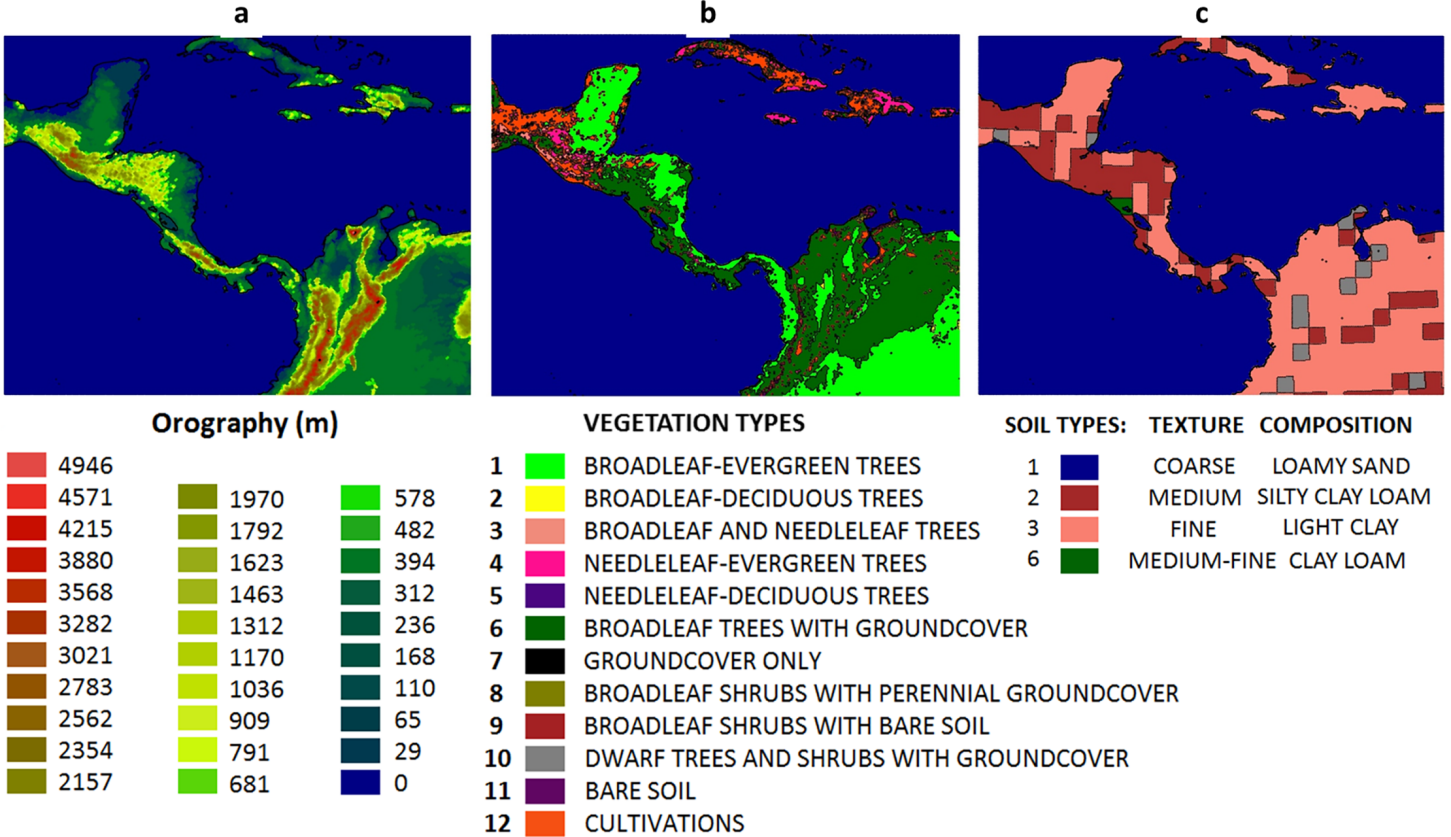
(a) Model topography (metres), (b) vegetation types, and (c) soil types at 8-km resolution.

The regional model is nested directly to the HadGEM2-ES state variables. These lateral boundary conditions are updated every 6 hours. The preparation for long-term integrations include tests of configuration by choosing the appropriate number of points, domain area, and number of vertical atmospheric levels. To allow the regional model to develop high-resolution atmospheric structure, a large domain is chosen. The model is set up at 8-km horizontal resolution and 50 vertical levels. Fig 1 shows the model topography, vegetation map [34], and soil map [35] at 8-km horizontal resolution in the model domain over Central America.

The simulation of the present climate period starts on 1^st^ January 1960 and ends in 31^st^ December 2005. The initial soil moisture and monthly mean sea surface temperature is taken from HadGEM2-ES. The monthly mean sea surface temperature from HadGEM2-ES is interpolated to daily values during the Eta model integration. The first year of simulation is discarded, and the baseline period is taken as the 30-year period between 1961 and 1990. Projections for the future climate start on the 1^st^ January 2006 and end in the 31^st^ December 2050. The future climate changes are assessed in the 30-year period 2021–2050. Equivalent CO_2_ concentrations are updated every 3 years according to the RCP4.5 scenario values.

### Evaluation of the baseline simulations

Various observational datasets were used for evaluating the downscaled simulations. Four datasets are used to evaluate mean precipitation: the Global Precipitation Climatology Project (GPCP) [36], taken for the period 1979–1990, the University of East Anglia-Climate Research Unit (CRU) [37], taken for the period 1961–1990, provided at 0.5^o^ latitude-longitude resolution grid, the NOAA CPC Morphing Technique (CMORPH) [38], taken for the period 1998–2013, at 8-km resolution grid, and the high spatial-resolution Climate Hazards Group Infra-Red Precipitation with Stations Data from the University of California in Santa Barbara (CHIRPS) [39], taken for the period 1970–1999, at 0.05° latitude-longitude resolution grid. These are 30-year period datasets, except for CMORPH data. Two temperature datasets, from CRU and produced by [40], are used to evaluate temperature. The average temperature derived by [40] will be referred to as Tavg from now on. All data are converted to monthly values.

## Results

We evaluated the baseline period to quantify the reliability of the downscaling projections for the region before assessing the future climate scenarios. After that, projections of climate change is assessed in terms of mean changes with respect to the baseline period and future tendencies of extreme climatic indicators. This is shown in the second part of the results. Two major features of Central American climate, simulation and projections for Mid-Summer Drought (MSD) and the Caribbean Low-Level Jet (CLLJ) are particularly analysed.

### Baseline

#### Seasonal mean

**Fig 2:**
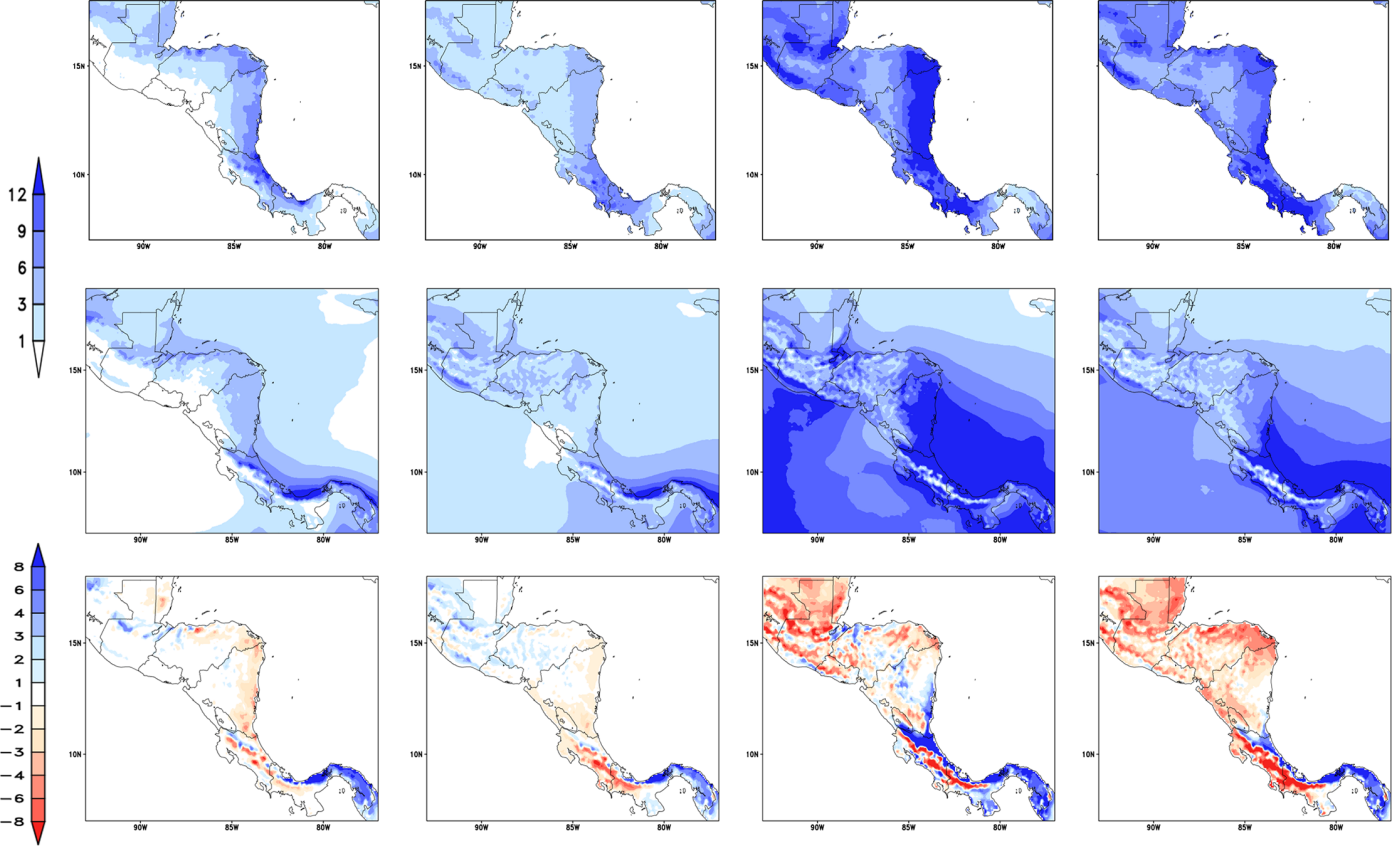
**Mean precipitation (mm/day), CHIRPS (top row), Eta-8km simulations (middle row) and differences between CHIRPS and Eta-8km (bottom row).** Average for the period 1961–1990 except CHIRPS (1970–1999), and for the seasons DJF, MAM, JJA, and SON (from left to right).

Fig 2 shows the seasonal climatological precipitation for the period 1961–1990. The top row shows the CHIRPS observations and the middle row shows the Eta model 8-km resolution (Eta-8km) downscaling driven by HadGEM2-ES global model simulations. The major feature of the observations is a wide band of high rainfall, which spans along the Caribbean lowlands from the east coast of Honduras up to Costa Rica and over central Guatemala. This feature is present throughout the year, being more intense during the rainy season from May to October. The Eta-8km model is able to simulate most of the precipitation characteristics, particularly during JJA. The intense precipitation region over the Caribbean coast, which mainly occurs during the winter (DJF) and summer (JJA), is well represented by the model. However, in general, simulated precipitation is underestimated with respect to observations in all seasons. Precipitation is overestimated on the east coast of Costa Rica and Panama, which may be associated with simulated precipitation over the ocean as the ITCZ approaches this region. The position and latitudinal displacement of the precipitation band associated with the ITCZ is well reproduced by the model along the year.

Table 1 shows the spatial correlation between the simulated precipitation pattern by the Eta-8km model and CHIRPS observations and the driver model, HadGEM2-ES. MAM is the season when the Eta-model precipitation exhibits the highest correlations. Results indicate that higher spatial resolution provides added value to the simulations over the coarse global model simulations.

**Table 1:** Pattern correlations between the Eta simulations and observations and between HadGEM2-ES simulations and observations, for DJF, MAM, JJA, and SON trimesters.

**Precipitation**				
Models\trimestre	DJF	MAM	JJA	SON
Eta 8km	0.70	0.57	0.58	0.52
HadGEM2-ES	0.47	0.54	0.35	0.61
**2-m Temperature**				
Models\trimestre	DJF	MAM	JJA	SON
Eta 8km	0.84	0.81	0.86	0.85
HadGEM2-ES	0.51	0.22	0.46	0.50

Precipitation observations used CHIRPS data and temperature observations used Tavg dataset.

**Fig 3:**
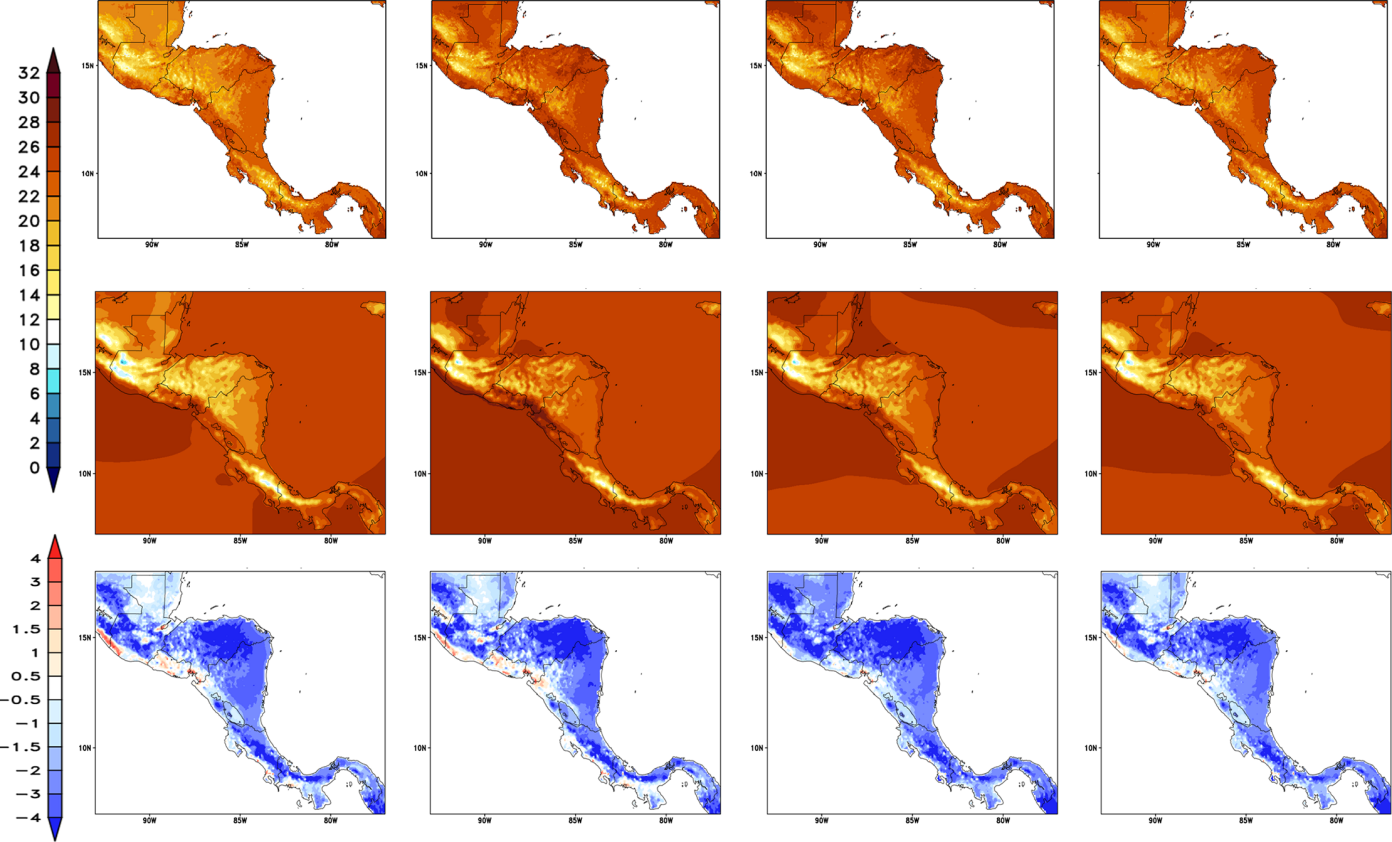
**Mean 2-metre temperature (°C) from Tavg [40] dataset (top row), Eta-8km simulations (middle row) and differences between CHIRPS and Eta-8km (bottom row).** Average for the period 1961–1990 except Tavg (1970–1999), and for the seasons DJF, MAM, JJA, and SON (from left to right).

The mean 2-metre temperature from the Tavg observations and Eta-8km simulations are shown in Fig 3. The model represents well the spatial patterns of 2-metre temperature for all seasons. The temperature gradient, the regional characteristics, and the seasonal variations are well reproduced by the model. The Eta-8km simulations captures well the areas of cold temperatures, such as in south Guatemala and south Costa Rica, and the areas of warmer temperatures such as in north Guatemala and coastal areas east of Honduras and Nicaragua. However, the model tends to underestimate temperatures almost everywhere in the domain, especially on high topography regions. The cold bias of temperature may be contributing to the underestimate of precipitation in the elevated areas as the convection trigger criterion in the Eta model depends on the near-surface air temperatures.

The pattern correlation between the simulated and the observed 2-metre mean temperature for the entire Central America is shown in Table 1. In all seasons, the spatial pattern of temperature is better reproduced by the regional model than by the global model used as lateral boundary conditions. The pattern correlation between the Eta model temperature simulations and Tavg observations starts from 0.86, whereas the correlation for HadGEM2-ES temperature starts from 0.51. JJA is the season when the Eta model 2-metre temperature simulations exhibit the highest correlations, but the skill in other seasons is comparable. The pattern correlation between Eta model simulations and CHIRPS observations are lower for precipitation than for temperature, but these correlations are still much higher than with HadGEM2-ES simulations, except for SON when the HadGEM2-ES correlates better with precipitation observations.

#### Annual cycle

**Fig 4:**
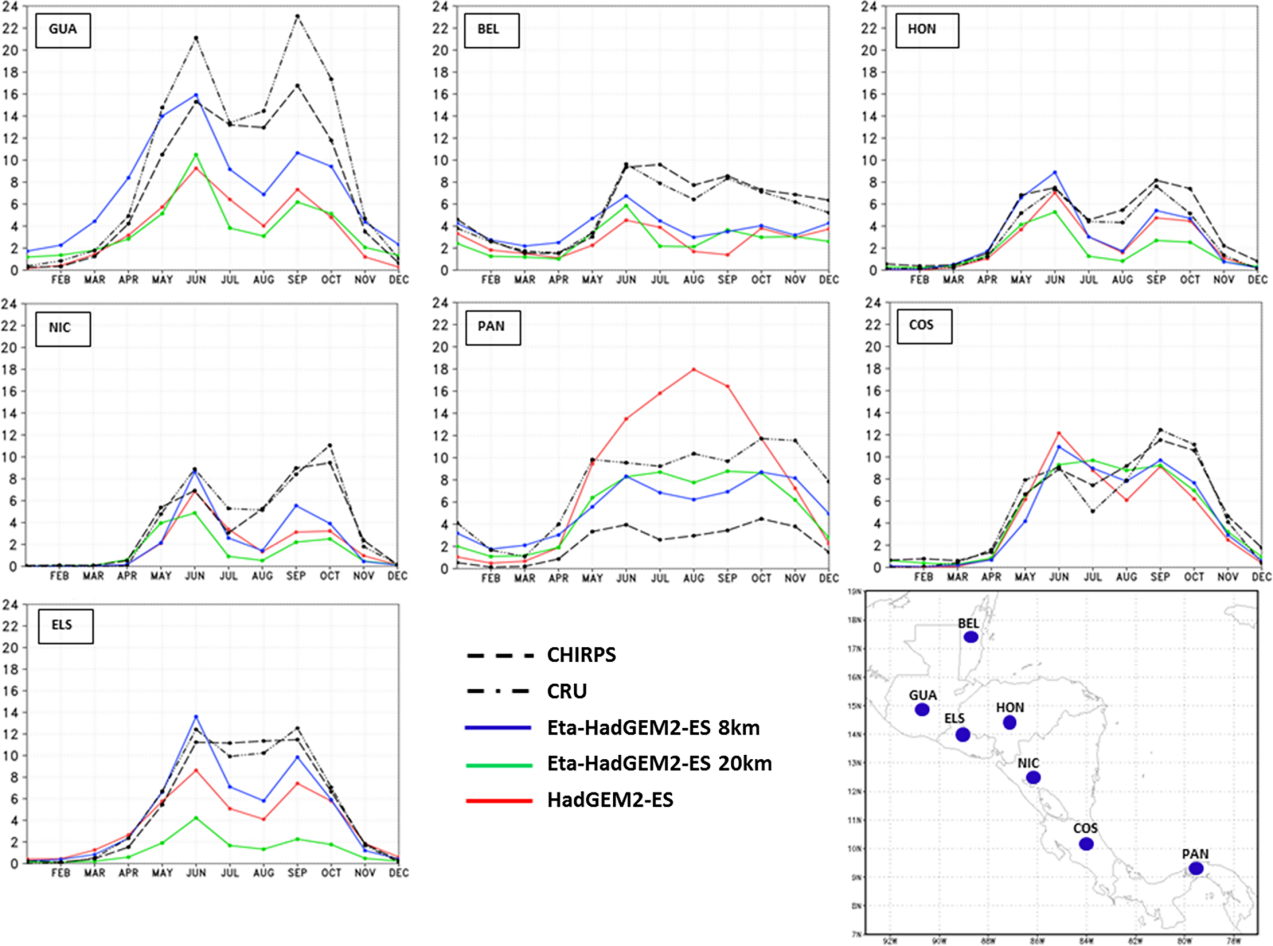
Mean annual cycle of precipitation (mm/day), averaged over the baseline 30-year period. Observations from CRU (dash-dot) and CHIRPS (dash) are plotted, as well as model simulations: Eta-8km (blue), Eta-20km (green), and HadGEM2-ES (red). The curves refer to the model grid-point that contains the capital city of the countries that identify each box: Guatemala (GUA), Belize (BEL), Honduras (HON), Nicaragua (NIC), Panama (PAN), Costa Rica (COS), and El Salvador (ELS).

Fig 4 shows the annual cycle of the observed and simulated precipitation for the capitals of Central America countries, Guatemala, Belize, Honduras, El Salvador, Nicaragua, Costa Rica, and Panama. The annual cycle is shown for two observational datasets, the CRU and CHIRPS, and for three model simulations, from the Global HadGEM2-ES, the Eta model at 20-km resolution, and the Eta model at 8-km resolution. The HadGEM2-ES serves as driver for both Eta runs as the global model output is used as boundary conditions. It is worth pointing to the large differences among the observational datasets.

During the driest months (DJF), the Eta simulations approach the CHIRPS observations. However, during the rainy season, the Eta simulations underestimate the precipitation with respect to CHIRPS observations, on almost all cities. The Eta model of higher resolution, 8 km, best reproduces the intensity of the precipitation annual cycle, which is compared against the Eta model at 20 km, and the driver HadGEM2-ES simulations. Therefore, in general, the higher resolution simulations added improvement over the coarser driver model simulations. The annual cycle of precipitation of the capital cities, which are located in the western portion of the continent, show a bimodal cycle of precipitation, with the two maximum rainfall peaks usually observed in June and September [41] and the reduction in rainfall between the two peaks, known as mid-summer drought (MSD) [42]. This variability is better reproduced by the Eta model at higher resolution, the 8 km. At this resolution, in general, the model captures the intensity of the two peaks of rainfall in most of the points. Similarly, for the capital cities to the eastern portion of the continent, the Eta model at 8 km best represents the annual cycle of precipitation. The onset of the rainy season, which usually occurs in June, is captured by the model simulations using higher resolution. In addition, the amount of rainfall is also well simulated by the Eta-8km simulations. In general, the first precipitation peak in the year seems better simulated than the second peak, which shows underestimated values.

**Fig 5:**
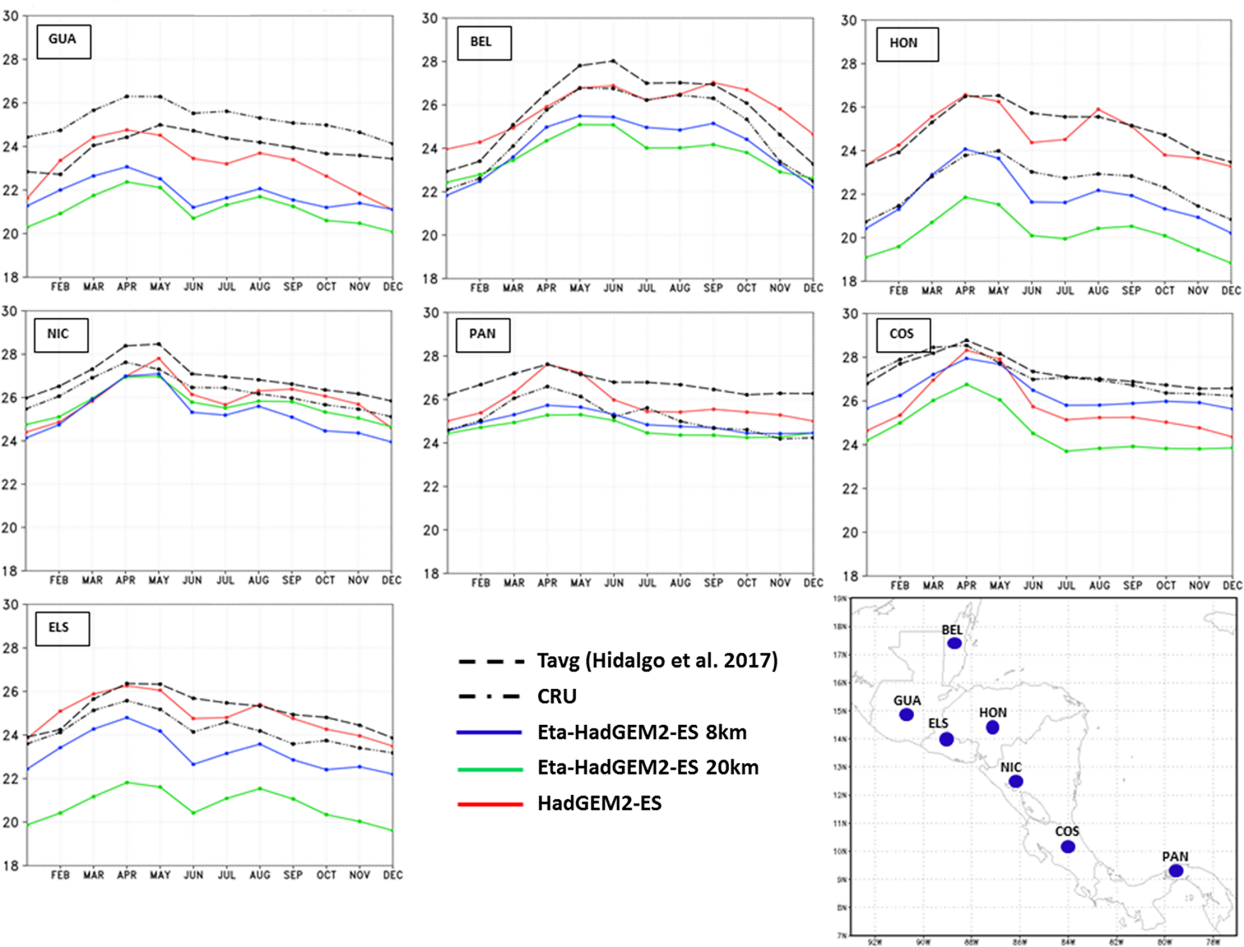
Mean annual cycle of 2-metre temperature (°C) averaged over the baseline 30-year period. Observations from CRU (dash-dot) and Tavg (dash) are plotted, as well as model simulations from Eta-8km (solid line and cross), Eta-20km (solid line and circle), and HadGEM2-ES (solid line and diamond). The curves refer to the model grid-point that contains the capital city of the countries that identify each box: Guatemala (GUA), Belize (BEL), Honduras (HON), Nicaragua (NIC), Panama (PAN), Costa Rica (COS), and El Salvador (ELS).

The mean annual cycle of 2-metre temperature for the capital cities is shown in Fig 5. The two observations show large differences in particular in Honduras’ capital. Honduras might be the capital with the most complex terrain The Eta-8km and Eta-20km simulations systematically show cold bias with respect to the CRU and Tavg from [40], resulting in an underestimate of the seasonal temperature cycle. The CRU is generally colder than the Tavg dataset. The temperature absolute errors in the Eta-8km simulations do not exceed 3°C on average. Despite the cold biases, the Eta-8km simulations are closer to the temperature observations than the Eta 20-km simulations. The shape of the annual cycle of the Eta model simulations follows closely the driver global model HadGEM2-ES temperature cycle. The HadGEM2-ES simulations reproduces the temperature better when compared with the regional model simulations, although some underestimate also occurs in some points.

#### Mid-Summer Drought

**Fig 6:**
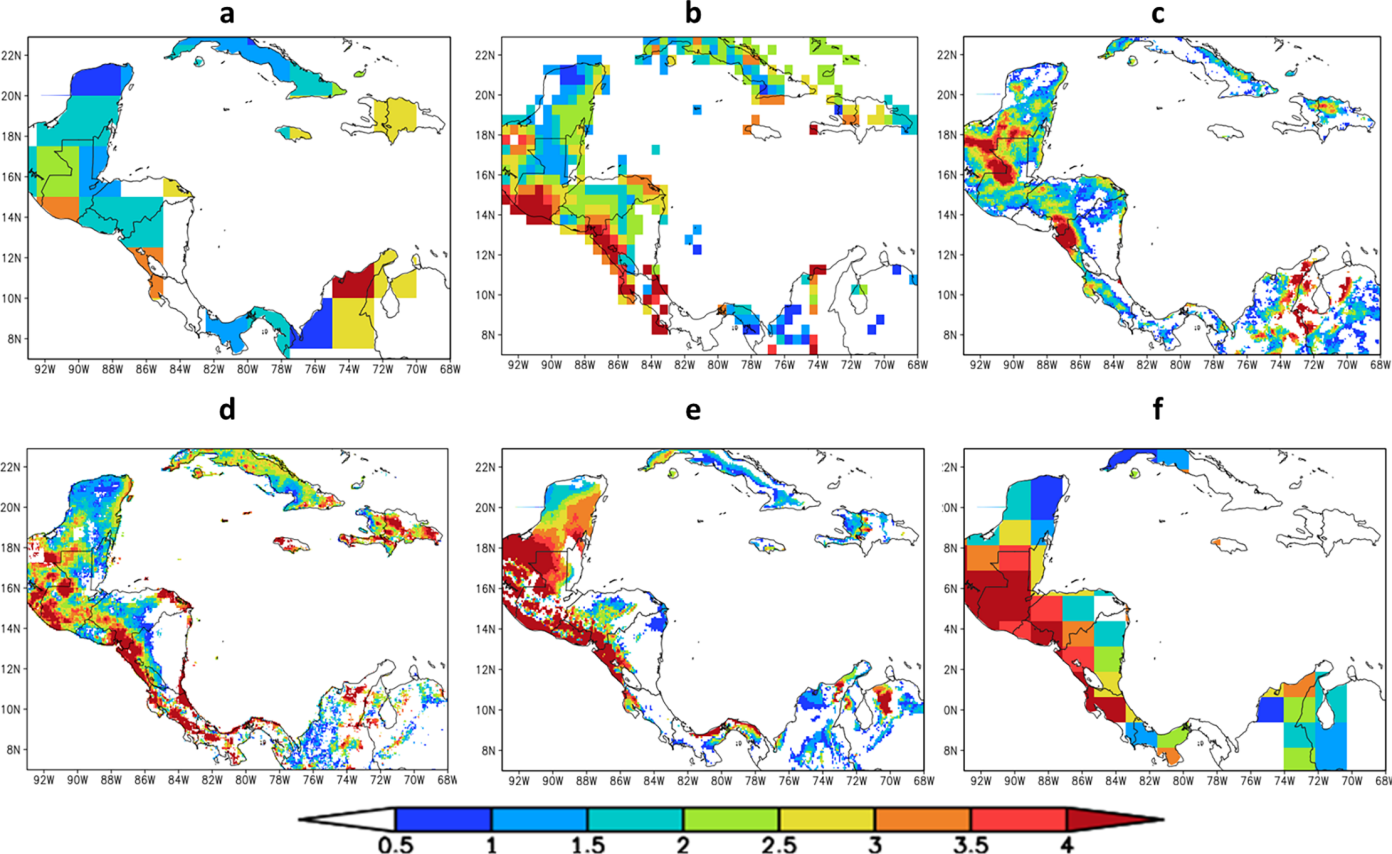
**Mid-Summer Drought strength (mm/day) calculated using different precipitation observational datasets:** (a) GPCP, (b) CRU, (c) CMORPH and (d) CHIRPS precipitation data, and using the baseline period (1961–1990) precipitation simulations from (e) Eta-8km and (f) HadGEM2-ES.

[43] developed an objective algorithm for the detection and estimating the strength of MSD. This algorithm uses monthly climatological precipitation data and does not assume a priori which months are maxima and which months represent the MSD. The algorithm is applied at every point of the dataset. Here, the algorithm is tested for GPCP, CRU, CMORPH, and CHIRPS observational datasets and these results are compared against HadGEM2-ES and Eta-8km baseline simulations (Fig 6). A Central America dry corridor, the ‘corredor seco’, starts in the north of Costa Rica and extends into Nicaragua, Honduras and Guatemala on the Pacific side and also includes a portion of the central Pacific coast of Panama, the Panama dry arch. CMORPH does not capture the dry arc over Pacific coast, which is clear over El Salvador. This area, with high intensity in MSD, is captured by the Eta-8km. All datasets miss the “dry arc” in Panama, except for HadGEM2-ES. The observational dataset show disagreement in some areas. This corroborates the large uncertainties in the observational datasets. In general, the spatial patterns of MSD estimated by Eta-8km and HadGEM2-ES simulations are reasonably well reproduced. The Eta-8km simulation shows better agreement in the intensity and position of observed MSD than HadGEM2-ES.

#### Caribbean Low-Level Jet

**Fig 7:**
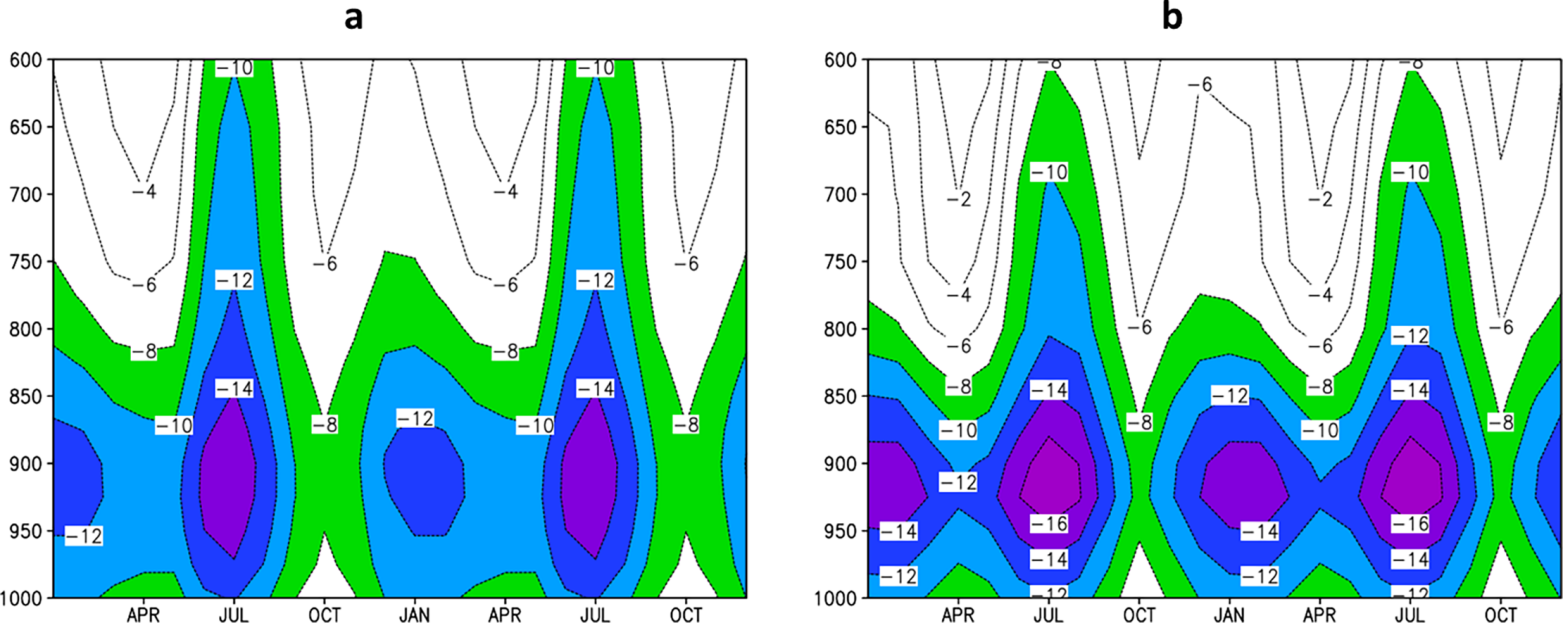
Annual cycle (repeated twice) of the vertical (1000–600 hPa) profile of zonal wind (m/s). Average over the CLLJ area (80–70°W, 12–16°N), for (a) CFSR Reanalysis data, and for (b) the Eta-8km simulation. Negative values refer to easterly winds.

The vertical profile of zonal wind is a suitable feature to investigate the model ability to represent atmospheric circulation in Central America. Fig 7 shows the annual cycle of the vertical profile of mean zonal wind over CLLJ area (80–70°W and 12–16°N) averaged over 1979–2008 period using CFSR reanalysis data [44] and over 1961–1990 period using Eta-8km simulations. The reanalysis shows the strongest jet in July and a secondary maximum in February. April and October are the weakest CLLJ months. This semi-annual feature of CLLJ is consistent with meridional gradient of sea surface temperature and the sea level pressure [45] The Eta-8km is able to capture the CLLJ semi-annual feature. The core of the jet at 925 hPa, with maximum in July and minimum in October, is also well reproduced by the high-resolution simulations. However, Eta-8km simulations show positive bias, about 2 m/s, in zonal wind velocity in comparison with CFSR in the layer between 950 and 900 hPa throughout the year, but show negative bias, about -2 m/s, above 700 hPa in July. The simulated CLLJ is not as deep as in the reanalysis data. The strength of the jet may have resolution dependency, as the errors are even larger when compared against the ERA40, a coarser reanalysis data.

### Projections

The assessment of future climate change are based on the projections produced by the downscaling of the Eta model at 8-km resolution, for the 30-year period between 2021 and 2050, under RCP4.5 scenario. In addition to mean features, we analyse trends of extreme climatic indicators.

#### Change in seasonal mean

**Fig 8:**
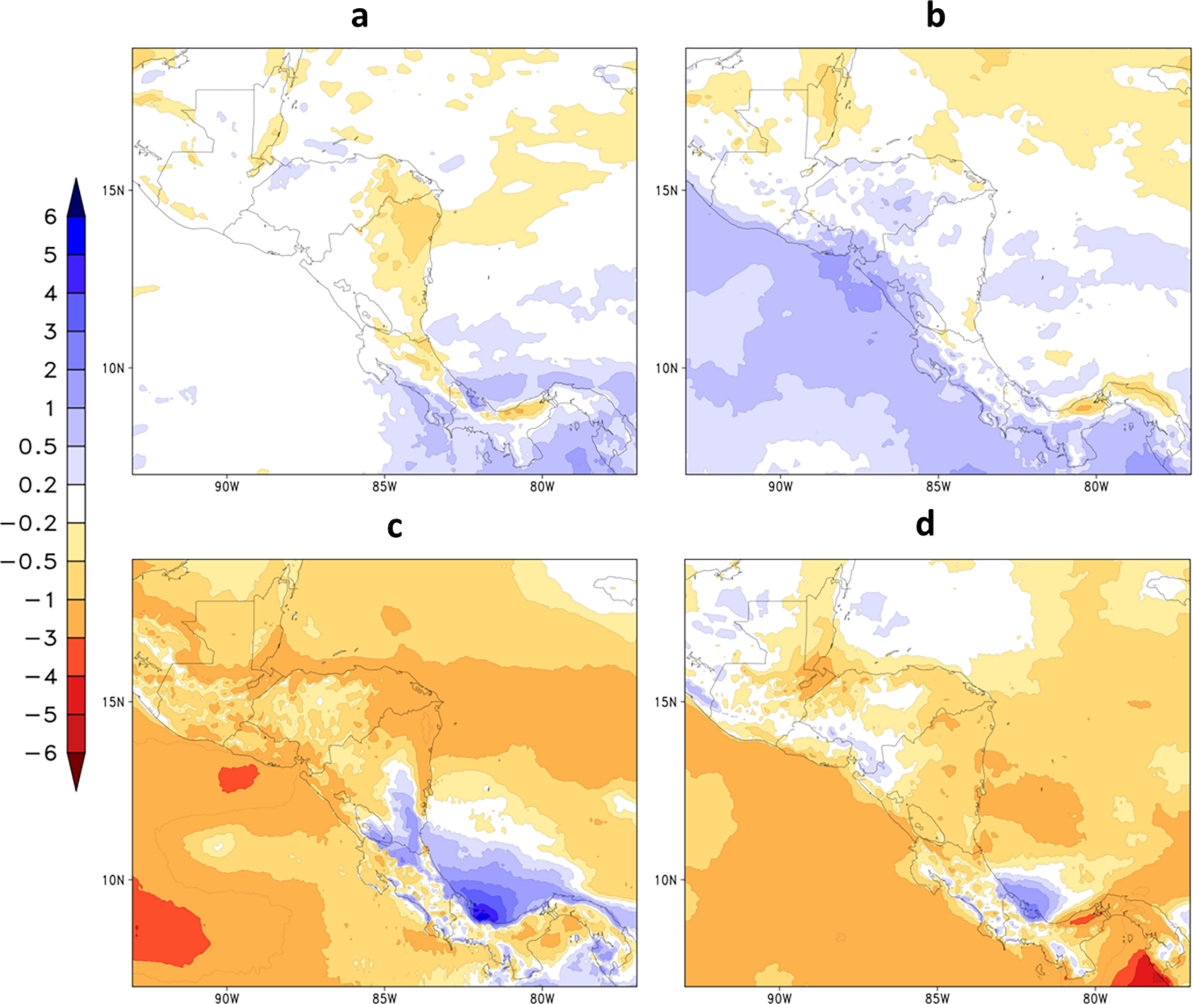
**Mean precipitation (mm/day) changes projected for the period 2021–2050, under RCP4.5 scenario, for (a) DJF, (b) MAM, (c) JJA, and (d) SON**.

Fig 8 shows projections of precipitation rate averaged for the future period, 2021–2050, and for each season under RCP4.5 emission scenario. The spatial distribution of projected precipitation is similar to the climatological pattern of the baseline period, 1961–1990 (Fig 2). However, precipitation decrease is projected over most of the continent in all seasons, but particularly in the rainy season. An analysis of end-of-the-century projections also show this drier signal over northern Central America with high agreement between GCM, and show disagreement over the southern part of the region over Panama and Costa Rica. An increase in precipitation is projected in MAM in some areas on the western part of the continent, such as south El Salvador up to Costa Rica and adjacent Pacific Ocean, and in JJA in southeast Nicaragua and along the eastern coast of Costa Rica and Panama. Between June and November, in JJA and SON seasons, precipitation is projected to decrease over most of the continent and adjacent Pacific and Atlantic Oceans, except near the eastern coast of Costa Rica and Panama, where a positive change in rainfall is expected. Over the Atlantic Ocean off the coast of Panama, the precipitation increase is projected to reach about 4 mm/day during JJA.

**Fig 9:**
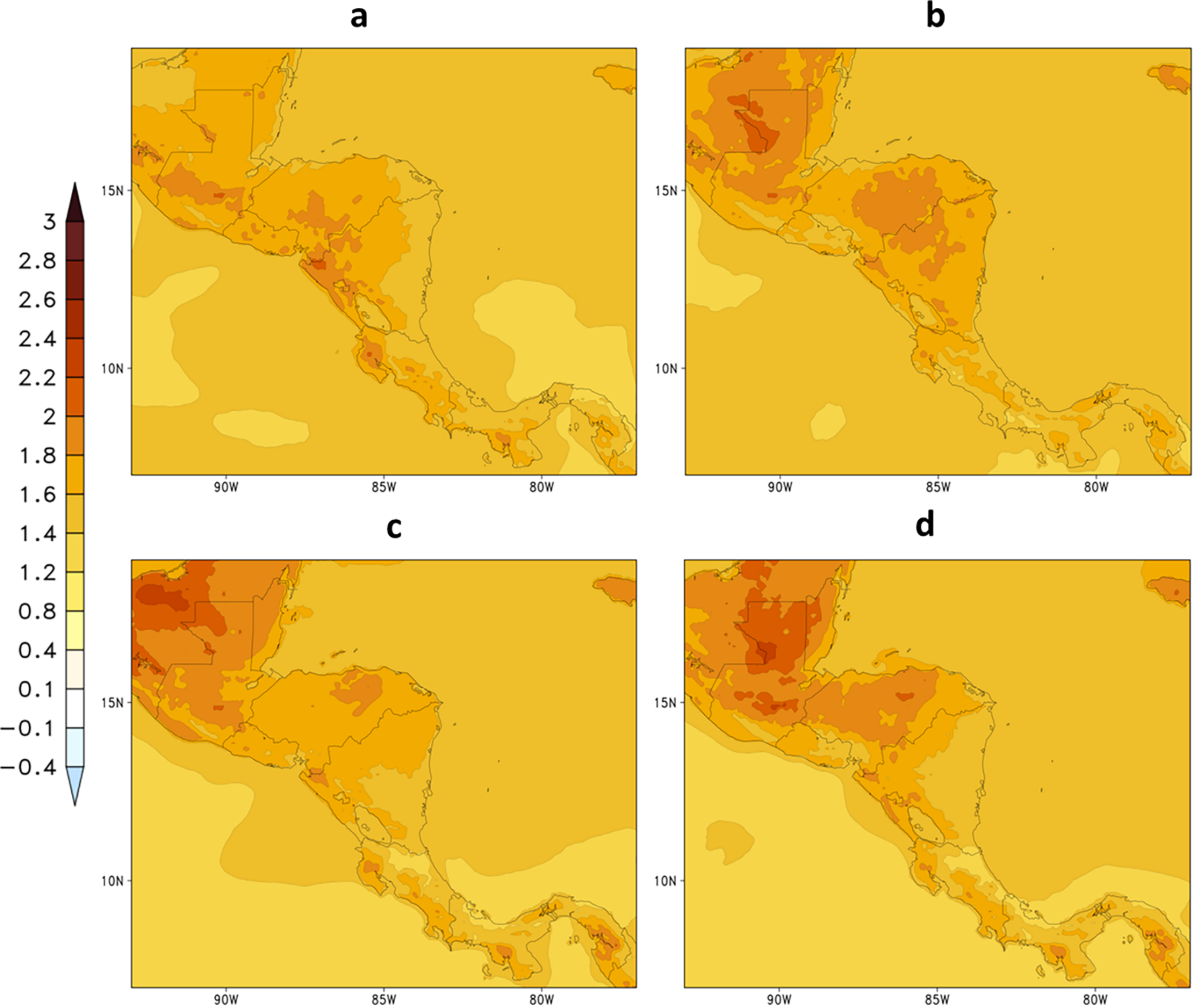
**Change in seasonal mean 2-metre temperature (^o^C) projected for the period 2021–2050, under RCP4.5 scenario, for (a) DJF, (b) MAM, (c) JJA, and (d) SON**.

Fig 9 shows projections of 2-metre temperature averaged over the future period, 2021–2050, and for each season under RCP4.5 emission scenario. Temperatures increase throughout the area, including the adjacent oceans. In comparison with the baseline period (Fig 3), stronger temperature increase is projected in the northernmost part of the region, where changes can range from about 1.8^o^ to 2.4°C. In the southern part of Central America, temperature increase ranges from about 1.6^o^ to 2.0°C. More intense warming is projected to occur in the SON season.

#### Changes in annual cycle

**Fig 10:**
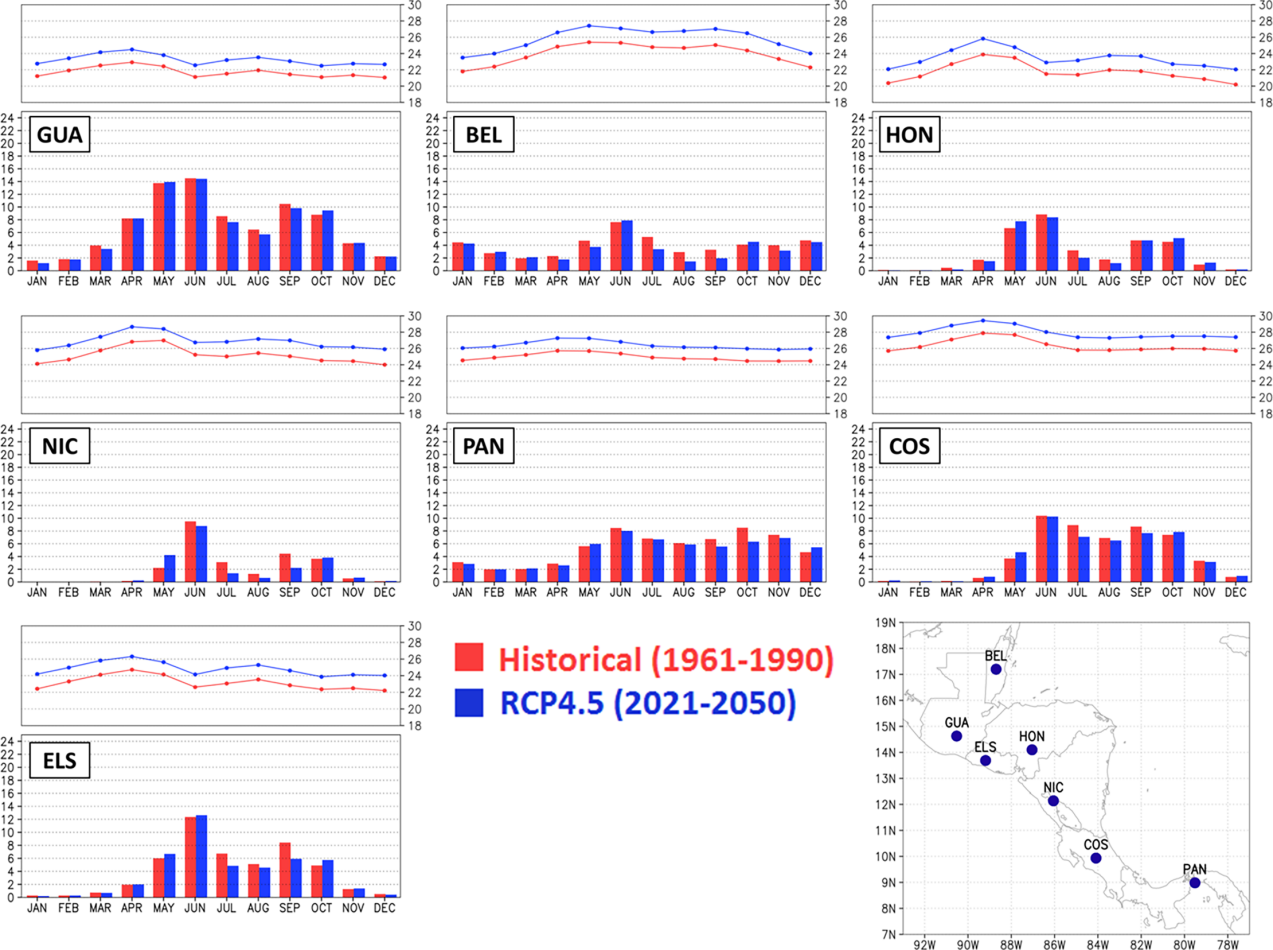
Annual cycle in precipitation (mm/day) and 2-metre temperature (^o^C) for the capital cities in Central America. Historical period (in red), 1961–1990, and future period (in blue), 2021–2050, under RCP4.5 scenario. The three initial letters of the country of the respective capital city identify each box: Guatemala (GUA), Belize (BEL), Honduras (HON), Nicaragua (NIC), Panama (PAN), Costa Rica (COS), and El Salvador (ELS).

Fig 10 shows the annual cycle of precipitation and 2-m temperature simulated by the model for the baseline period (1961–1990) and future (2021–2050) for the capital city points. Although in some months it Is projected an increase in rainfall in the future period, projections for all model grid points that contain the capital cities show reduction of precipitation, especially between the months of June to October, when the mid-summer drought events generally occur. This reduction in future precipitation during mid-summer drought period is clearly seen in the annual cycle of Costa Rica and Guatemala. In addition, the annual cycle of future precipitation remains close to the present, such that the future wettest and driest months remain the same as in the baseline climatic period. Similar to precipitation, the annual cycle of future temperature remains close to the present. However, a mean increase in temperature of about 2°C is projected in all city points.

#### Changes in the Mid-Summer Drought

**Fig 11:**
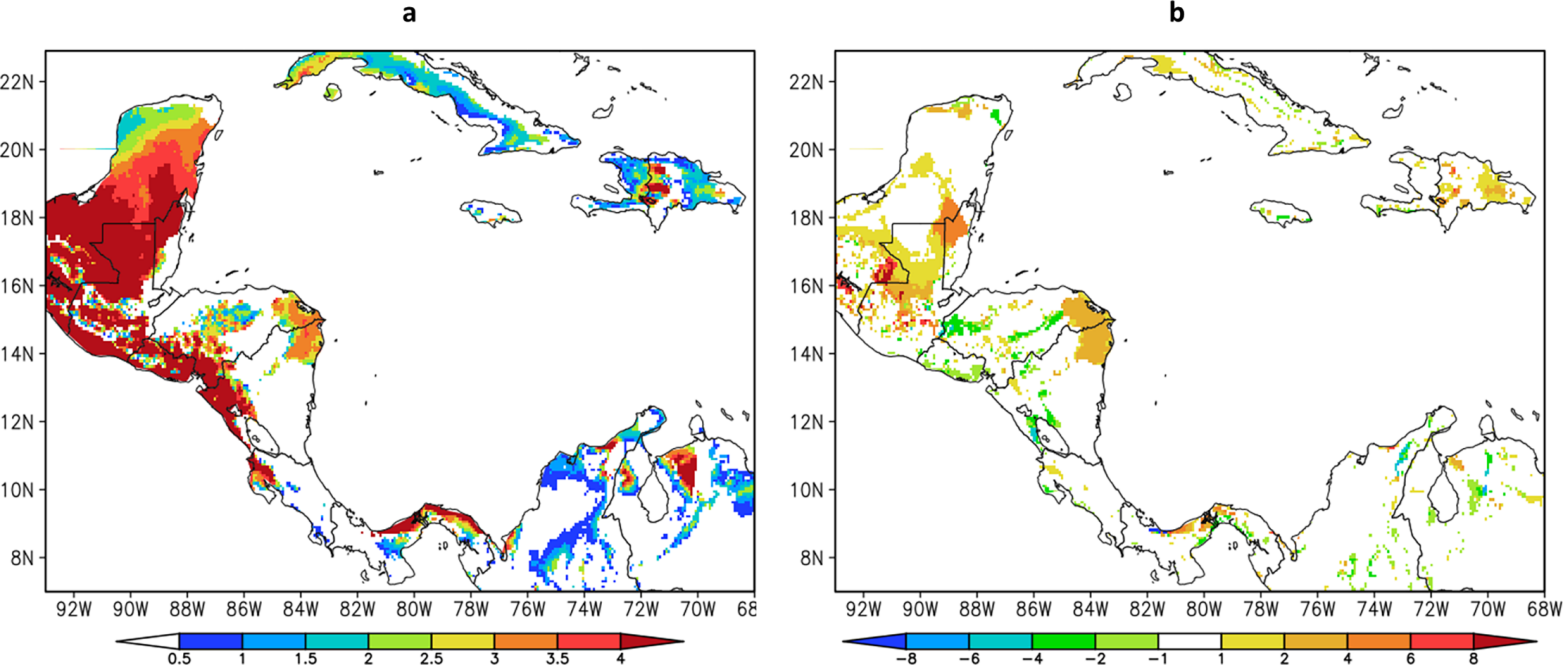
(a) Mid-Summer Drought (MSD) strength (mm/day) projected for 2021–2050 by Eta Model at 8km and (b) MSD difference (mm/day) between 2021–2050 and 1961–1990.

Projected changes in Mid-Summer Drought (MSD) for the future period 2021–2050 are shown in Fig 11. The downscaling projections under RCP4.5 scenario of the MSD shows similar spatial pattern to the present climate simulation. However, the MSD intensifies over Guatemala, northwest Belize, east Honduras, northeast Nicaragua and a narrow strip along the Atlantic coast of Panama. [12] finds a similar trend over northern Central America over most of El Salvador, Honduras and southern Guatemala based on GCM ensemble analysis. These regions already exhibit strong MSD in the present climate simulations, but the projected MSD strength increases from about 2 mm/day to 6 mm/day in RCP4.5 scenario. The increase in MSD strength is mostly due to stronger precipitation reduction in the months of minimum precipitation within the MSD season as shown in the annual cycle of precipitation (Fig 10). Some grid points, where MSD do not occur during the baseline period, may develop an MSD under RCP4.5 scenario.

#### Changes in the Caribbean Low-Level Jet

**Fig 12:**
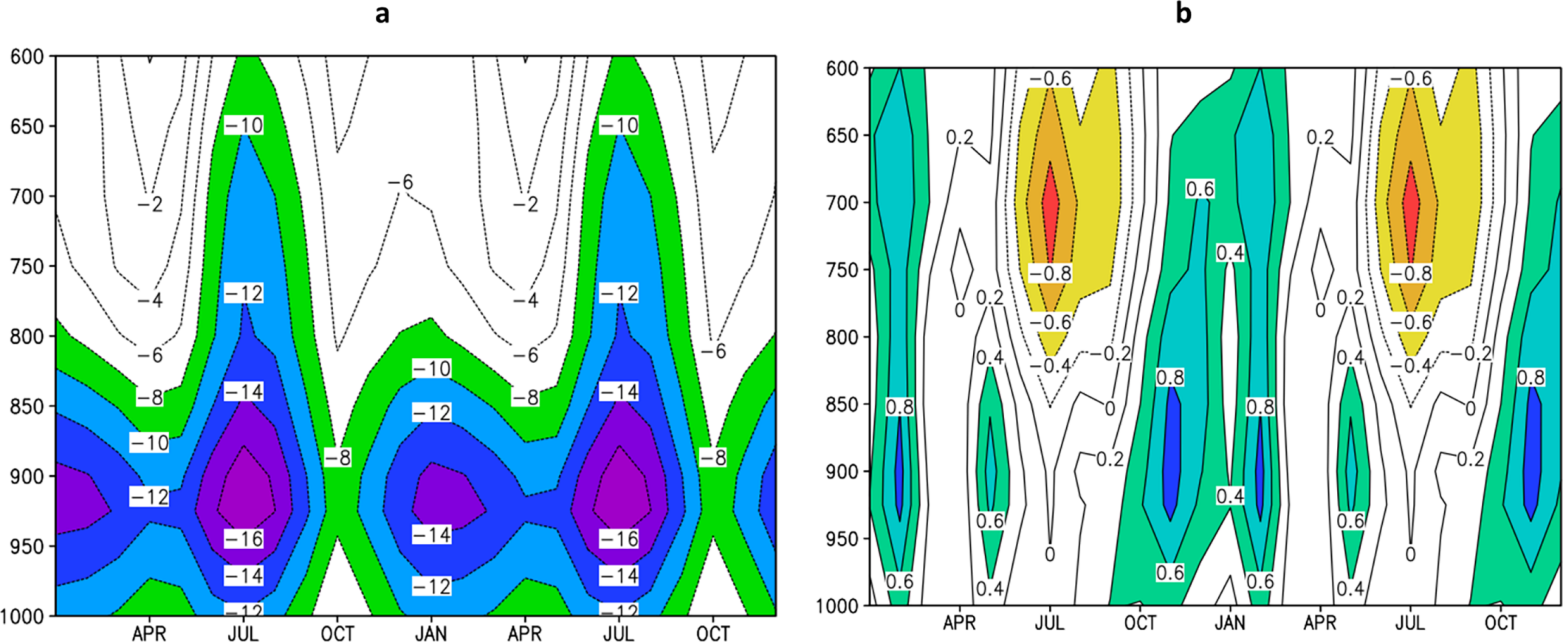
Annual cycle (repeated twice) of the vertical (1000–600 hPa) profile of zonal wind (m/s). Average over the Caribbean Low-Level Jet area (80–70°W, 12–16°N) projected for (a) 2021–2050 and (b) difference of zonal wind between 2021–2050 and 1961–1990 period. Negative values in (a) refer to easterly winds, whereas negative values in (b) indicate strengthening (orange shading) of easterly wind velocity.

The changes in CLLJ projected by the Eta model simulations at 8 km are shown in Fig 12. In general, the climate change causes little impact on the CLLJ. The projections suggest some weakening in the CLLJ in the layer between 1000 hPa and 600 hPa from November to February, and some strengthening in the layer between 800 hPa and 600 hPa from June to August (Fig 12B). The wind in 925 hPa, where the core of CLLJ is located, weakens during the entire year in these projections for the period 2021–2050. However, toward the end of the century, [46] assessed the CLLJ at this same level, 925 hPa, and found strengthening of the winds from May to November based on PRECIS projections for A2 and B2 scenarios. This disagreement in the trends of the CLLJ strength leads to little confidence on the possible changes on the CLLJ. [47] showed using ERA-40 reanalyses that when the CLLJ is strong, precipitation is reduced in the Caribbean Basin. However, the jet is slightly weaker and precipitation in the Caribbean region has reduced in these projections.

#### Trends in climatic extremes

**Fig 13:**
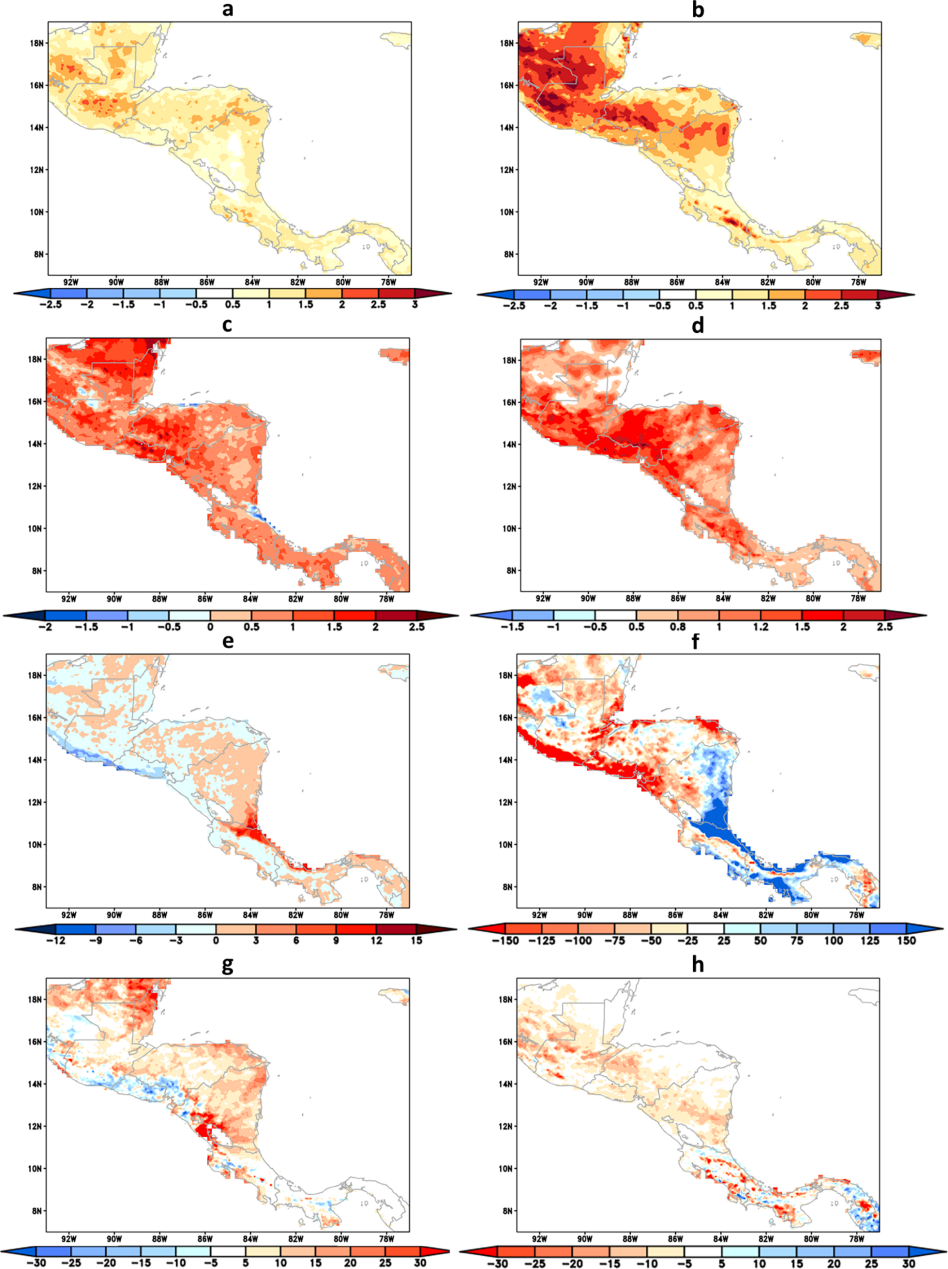
Trend of climatic extreme indicators. **(**a) TNx (°C), (b) TXx (°C), (c) TNn (°C) and (d) TXn (°C), (e) R50mm (days), (f) R95p (mm), (g) CDD (days), (h) CWD (days), within the period 2021–2050. The ocean areas are masked out.

The linear trends of the extreme climatic indicators are shown in Fig 13. The positive/negative trend indicates increase/decrease within the 30 years, between 2021 and 2050, in extreme climatic conditions.

The trends of temperatures of warm nights and warm days, indicated by TNx and TXx, respectively, are projected to increase everywhere in the domain in the future period of 2021–2050. Stronger warming rates are shown over the northern portion of the continent, in particular over the eastern slopes of the mountains in Guatemala, Honduras, and Nicaragua. Similarly, the trends of temperatures of the cold days, indicated by TXn, increase everywhere, but at stronger warming rate over the Pacific coast of Nicaragua, El Salvador, Honduras and Guatemala.

The trend of R50mm index provides information on precipitation exceeding 50 mm/day, which represents heavy rains. The positive trends of R50mm are mostly found in the Atlantic coast of Costa Rica and northern parts of Panama, whereas negative trends are more clearly found along the Pacific coast of El Salvador and Guatemala. The R50mm shows some similarity with 90^th^ percentile of precipitation index, R90p, but this latter has much stronger signal. R90p refers to extreme precipitation, rains heavier than R50mm, which occur less frequently. The R90p indicator shows strong increase in the Atlantic coast of Panama, Costa Rica, and Nicaragua. The Pacific coast of Panama and Costa Rica also shows some increasing rate, but smaller. In the Pacific coast of El Salvador, Honduras, and Guatemala we found a decreased tendency of extremely heavy precipitation, indicated by R90p, maybe related to the decrease of winds or hurricane activity.

Consecutive dry days indicator, CDD, could be used as a proxy for the extension of the dry season, which helps to assess potential demand of water for irrigation or the needs for stricter reservoir management in order to face long water deficit periods. The trends show no clear change around Panama, Costa Rica and Nicaragua. Positive trend is found farther north, mostly on the eastern slopes of the Andes Mountains. Negative trends are found over El Salvador and along the Southern coast of Guatemala. Therefore, these areas are not likely to suffer drier conditions than present time. These areas of decreasing CDD are not necessarily the areas of increasing consecutive wet days, CWD. The positive trend in CWD is found in isolated areas in Panama and Costa Rica where negative trend in CDD also occurs indicating increased climate variability. Most of the negative trends in CWD are found in the interior of Nicaragua, Honduras, and Guatemala.

**Fig 14:**
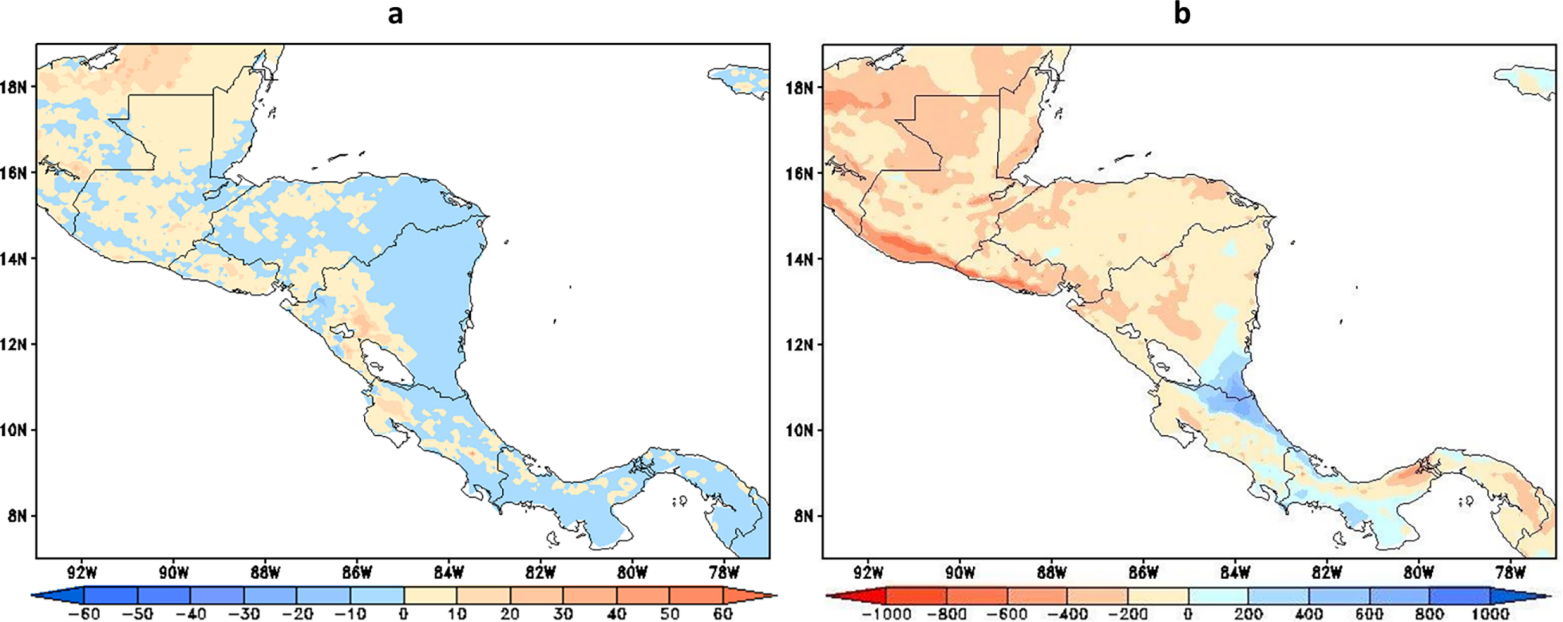
(a) Trends in dry spells, defined as annual largest number of consecutive days when ET < = 0.5*ETP and (b) trends in annual P-ETP in mm year^-1^, within the period 2021–2050.

Dry spells affecting vegetation growth can be assessed through the relationship between actual (ET) and potential (ETP) evapotranspiration [48], which combines the effects of precipitation, temperature, soil moisture, and vegetation cover, among other atmospheric conditions. Fig 14A shows the trends in the maximum number of consecutive days per year when ET < = 0.5 ETP. Negative trends are shown for most of the areas in Panamá and Costa Rica and for the Atlantic basin in Nicaragua and Honduras. On the other hand, over Pacific basin in Nicaragua and El Salvador, and most of Belize and Guatemala trends are positive suggesting an increment in water deficit events in these regions. This pattern of trends resembles the pattern of evapotranspiration changes shown by [49]. In addition, annual mean water excess, P-ET, is shown as a proxy of water availability for runoff in the region. According to Fig 14B, P-ET increases during the 2021–2050 period in the lower parts of the Atlantic basins of Costa Rica, southeast Nicaragua and the Pacific basins of Panamá. [49] found a regional decrease in water availability using an biogeography model under an ensemble of future GCM runs by the end of the century.

## Conclusions

Simulations of the present and future climate of Central America derived from the HadGEM2-ES were downscaled to 8-km resolution using the Eta Regional Climate Model. The future simulations adopted the RCP 4.5 scenario. The simulations were carried for the period between 1960 and 2050. The evaluation of the baseline climate simulations showed that the general spatial patterns of precipitation and temperature are reasonably captured. However, the simulations underestimate the precipitation during the wet season. Simulations also show cold bias in the domain. The high-resolution clearly show advantage over the coarse driver global model in simulating the baseline climate. Mean warming of about 1 and 1.5 ^o^C are projected for the future period between 2021 and 2050 for the region. Precipitation reduction is projected for the rainy season as well as the strengthening of the Mid-Summer Drought, which can have implications to agriculture and energy production. High-resolution projections indicate warming of extreme temperatures. Extreme precipitation in general shows decreasing trend in the northern part of the continent and increasing trend in the southern parts, in particular in Costa Rica and Panama, for the future period. This resembles the dry spell trend. In most of the countries in Central America, water availability shows negative trend in these projections, except in the eastern coast of Costa Rica and western coast of Panama. These trends are in agreement with the general precipitation reduction projected for the region.

Despite the use of a single emission scenario, from a single global climate model, the results shown here provide more detailed information than global models due to the high resolution of these simulations. The narrowness of the continent with high mountains limits the use of coarse resolution global model simulations for the region. This work provided a unique dataset of very high resolution to investigate the impacts of climate on various socio-economic sectors in the region.
